# Epidemiological Scenario of Dengue in Brazil

**DOI:** 10.1155/2015/321873

**Published:** 2015-08-30

**Authors:** Rafaelle C. G. Fares, Katia P. R. Souza, Germán Añez, Maria Rios

**Affiliations:** United States Food and Drug Administration, Center for Biologics Evaluation and Research, Silver Spring, MD 20993, USA

## Abstract

Dengue is the most important reemerging mosquito-borne viral disease worldwide. It is caused by any of four *Dengue virus* types or serotypes (DENV-1 to DENV-4) and is transmitted by mosquitoes from the genus *Aedes*. Ecological changes have favored the geographic expansion of the vector and, since the dengue pandemic in the Asian and Pacific regions, the infection became widely distributed worldwide, reaching Brazil in 1845. The incidence of dengue in Brazil has been frequently high, and the number of cases in the country has at some point in time represented up to 60% of the dengue reported cases worldwide. This review addresses vector distribution, dengue outbreaks, circulating serotypes and genotypes, and prevention approaches being utilized in Brazil.

## 1. Introduction

Dengue is an arthropod-borne disease caused by the* Dengue virus (DENV)* [1]. DENV is an enveloped, single-stranded, positive-strand RNA virus, member of the genus* Flavivirus* in the Flaviviridae family. It is transmitted from human to human through the bite of mosquitoes of the genus* Aedes* [2]. DENV transmission by transfused blood is rare; however it indeed occurs and has been documented in Brazil [3].

There are four distinct but antigenically related types or serotypes of DENV (DENV-1 to DENV-4). The outcomes of infection by any of the DENV types range from asymptomatic, subclinical to symptomatic infections [4–7]. Symptomatic infections vary from a mild, flu-like illness known as dengue fever (DF) to a life-threatening form called severe dengue (SD) [8]. Severe dengue was previously known as dengue hemorrhagic fever (DHF)/dengue shock syndrome (DSS) [8].

The onset of DF is sudden, characterized by high fever accompanied by intense frontal headache, fatigue, retroorbital pain, myalgia, arthralgia, and rash. SD is defined by an increase in vascular permeability (“plasma leakage”), hemorrhagic manifestations, and decreased platelet counts near the time of defervescence, which may progress to clinical hypotension and shock [6, 9–11]. In the absence of supportive care, SD fatalities may occur in approximately 4% of cases [1, 10]. Thrombocytopenia is commonly observed in both DF and SD as a result of platelet activation and apoptosis, triggering platelet clearance [5, 12].

To date, there is no effective vaccine or specific antiviral therapy for dengue. Early diagnosis and appropriate management of SD, including hospitalization, prevent fatalities [13]. Laboratory diagnosis of dengue is based on the detection of the virus and antibodies to the virus or combined detection of antigens and antibodies by serology (detection of specific IgM and IgG anti-DENV) [14], detection of the NS1 antigen of the virus, virus isolation in susceptible mosquitoes or in mosquito or mammalian cell lines, and nucleic acid detection tests (end-point PCR, TaqMan, and other nucleic acid detection assays) [15–18]. The detection of NS1 using ELISA is a sensitive method (>90%) for identification of primary DENV infections. In secondary infections, the sensitivity ranges between 60 and 80% [19, 20]. However, the test is not sensitive enough for blood donor screening. Some NS1 ELISA has low sensitivity for DENV-4 and it has contributed to the failure of reporting epidemiological data concerning DENV-4 cases in Brazil [21, 22].

While there has been no definitive association of the distinct DENV types with clinical course of disease, there are reports suggesting that DENV-2 and DENV-3 cause severe disease more frequently than the other serotypes and that DENV-4 causes a milder illness [23, 24]. DENV-2 was the most prevalent serotype in several outbreaks in the Americas [6] and was epidemiologically classified as the most relevant serotype worldwide due to its association with the highest number of intense outbreaks, followed in sequence by DENV-3, DENV-1, and DENV-4 [25].

Knowledge about the pathogenesis of dengue infection is limited, but both viral and immune host factors appear to be associated with severe illness [26]. Nevertheless, the involved DENV type and the host genetic makeup may explain some differences in clinical manifestations observed, since genetic polymorphisms appear to provide protection or predispose to more severe forms of dengue [27].

Studies carried out in epidemic regions led to the generation of hypotheses about the development of SD [28]. They include antibody-mediated pathogenesis [4], host cytokine storms [29], host genetic factors [24], characteristics of virus isolates [30–32], viral load in the acute phase [33, 34], and the host nutritional status [35]. DENV can interact with dendritic cells, monocytes/macrophages, hepatocytes, and endothelial cells leading to the release of immune mediators during SD [31, 36, 37]. However, how the production of these cytokines is induced and their role in dengue pathogenesis are still not clear.

The global dengue pandemic appears to have begun in the Asian and Pacific regions where the first epidemic of dengue was reported, in 1779-1780 [38]. Ecological changes occurring since that time probably favored the geographic expansion of the vector and its increase in density. The high number of susceptible individuals (local populations, soldiers) and their widespread movement probably created conditions that facilitated the dispersion of the viruses [39, 40]. A number of complex factors contributed to the emergence and reemergence of dengue, as, for instance, population growth and unplanned urbanization associated with poverty and health inequality [40, 41].

The number of cases of DF and SD has steadily increased worldwide, and dengue infections have spread to new areas of the world, such as North America and Europe [42]. The disease is endemic in more than 100 countries, and the World Health Organization (WHO) estimated that over 40% of the world's population is at risk of dengue, with 50–100 million dengue infections reported worldwide every year [1]. However, Bhatt et al., 2013 [43], using new modelling approaches, estimated that 390 million dengue infections (apparent and unapparent) occur per year, three times higher than previous WHO estimates.

In the Americas, 1,173,248 suspected dengue cases were reported in 2014, of which 341,192 were confirmed by laboratory tests; 16,008 cases were reported as severe dengue and 684 deaths occurred due to the infection [44]. As demonstrated in a recent review, Brazil presented the fifth highest incidence of DF among Latin American and Caribbean countries from 1995 to 2009 [45]. Between the countries of the Southern Cone—Argentina, Brazil, Chile, Paraguay, and Uruguay—Brazil had the highest incidence rate of dengue: 294.02/100,000 inhabitants in 2014 [44].

**Figure 1:**
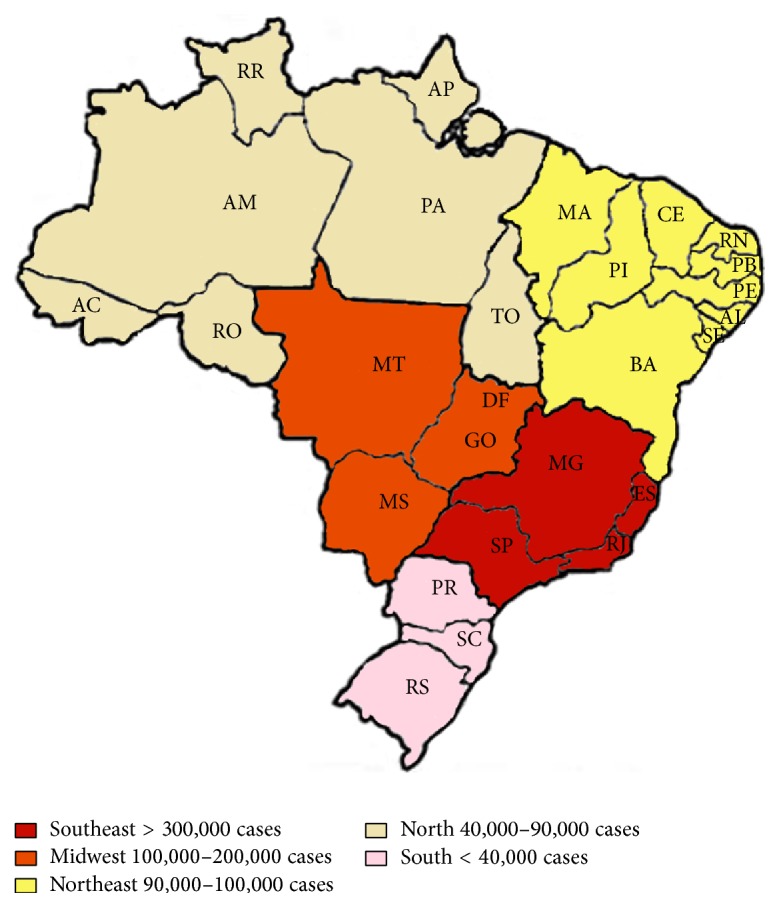
Number of dengue cases reported by the Brazilian Ministry of Health in 2014 distributed by geographical region. Brazil's map is divided according to the regions and number of dengue cases. The numbers were obtained from Brazilian Ministry of Health website, accessed at http://portalsaude.saude.gov.br/. Regions and their states are listed in Table 1.

**Figure 2:**
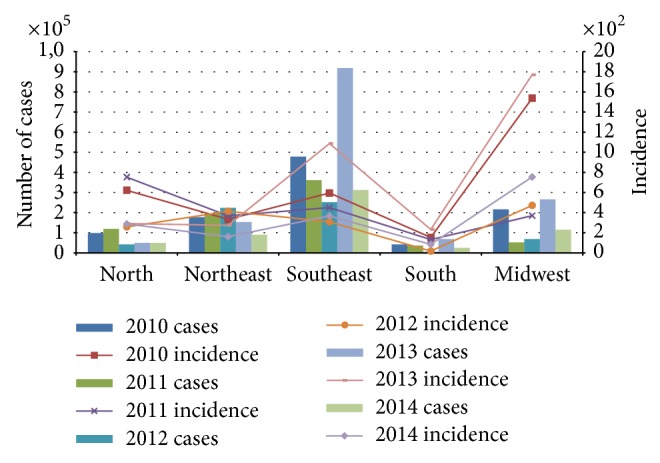
Number of cases and incidence/100,000 inhabitants reported by the Brazilian Ministry of Health in the last 5 years (2010–2014). The numbers were obtained from Brazilian Ministry of Health website, accessed at http://portalsaude.saude.gov.br/.

Brazil is composed of 5 geographical regions, shown in Figure 1 and Table 1. In 2014, the Southeast region reported the majority of suspected cases in the country (312,318 cases; 52.8%), followed by the Midwest (114,814 cases; 19.4%), Northeast (90,192 cases; 15.3%), North (49,534 cases; 8.4%), and South (24,222 cases; 4.1%) (Table 1; Figure 1). This geographical trending has been previously observed, with the Southeast region presenting the highest number of cases and the South region the lowest ones (Figure 2). However, when comparing incidence/100,000 inhabitants, the Midwest region presents the highest numbers (Figure 2) due to population size (Table 1). Only a few states had an increase in the number of cases in 2014 compared to 2013: Acre, Tocantins, São Paulo, and Distrito Federal. Between the 10 municipalities with higher number of cases reported, 5 are located in the state of São Paulo [46].

Brazil is considered a tropical country in its entirety because of its hot and humid climate which provides a receptive and highly favorable environment for proliferation of the dengue vector. The different climate zones have differences in the rain dynamics in the areas of the coastal strip, altitude variation, and so forth. DENV activity occurs throughout the year, but the majority of outbreaks and the highest levels of vector infestation show a marked seasonal pattern, occurring during the Brazilian rainy season, from December to May, which are also the hottest months of the year [47].

The scope of this review includes the epidemiological situation of dengue in Brazil, the temporal-spatial distribution of outbreaks, serotype and genotype distribution, geographical distribution of vector, and an analysis of measures that have been or could be taken to address this problem in the country.

## 2. Dengue in Brazil

### 2.1. Epidemiology and Outbreaks

**Figure 3:**
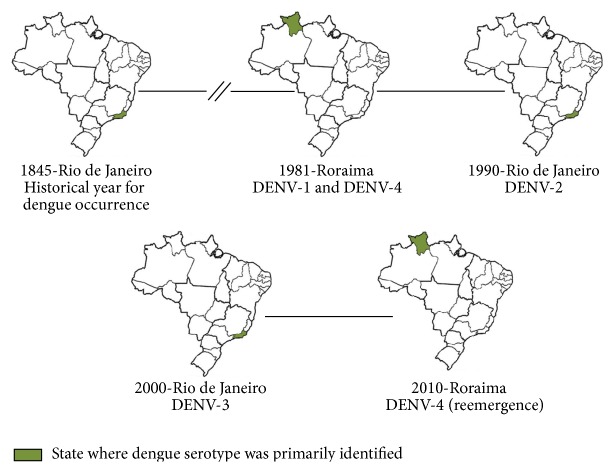
Timeline of introduction of dengue serotypes in Brazil. The states where dengue serotypes were primarily identified are highlighted in green. The figure was assembled with information collected from literature as described in the paper.

Dengue has been present in Brazil since 1845, when the first epidemic was reported in the state of Rio de Janeiro (Figure 3) [48]. Other epidemics were registered during the years 1851–1853 and 1916–1923 [49]. The mosquito eradication program to prevent urban yellow fever, coordinated by the Pan American Health Organization (PAHO), functioned successfully to keep Brazil free of* Aedes aegypti* until 1976. After that, the first evidence of a dengue epidemic was in 1981, when dengue cases occurred due to reinfestation of urban areas of Brazil by* Ae. aegypti* [47, 48]. This epidemic took place in the state of Roraima (Figure 3) and was caused by DENV-1 and DENV-4 (Table 2) [50]. It was the first laboratory and clinically reported dengue outbreak in Brazil with the presence of both serotypes [48, 50]. Nevertheless, the disease only received proper attention in 1986 and 1987, after DENV-1 was introduced into Rio de Janeiro [6, 47, 51, 52] (Figure 3). More than a million individuals from Rio de Janeiro were infected with DENV-1 [52]. DENV-1 was also responsible for epidemics in Ceará and Alagoas states in 1986 and in Pernambuco state in 1987 [53].

DENV-2 spread across the country after its first identification in 1990 in the state of Rio de Janeiro (Figure 3), where the first cases of SD were documented [26, 47, 52, 54] and 8 deaths due to SD were registered [46]. Both DENV-1 and DENV-2 have been possibly introduced in Brazil from Africa [49].

During the 1990s, the spread of DENV-1 and DENV-2 intensified and outbreaks affected Southeast and Northeast states [55–57] (Table 2). A total of 1,696 and 7,374 cases of dengue were reported in 1992 and 1993, respectively, but no deaths were attributed to dengue in these years. All cases occurred in the Southeast region with exception of the state of Espírito Santo [46].

In 1994, 12 states of Brazil documented cases of the disease: Tocantins, Piauí, Ceará, Rio Grande do Norte, Alagoas, Bahia, Rio de Janeiro, São Paulo, Mato Grosso do Sul, Mato Grosso, Goiás, and Distrito Federal, with the highest incidence rate in the Northeast region (112.2 cases/100,000 inhabitants). During the 1990s, the highest incidence rate of dengue (313.8 cases/100,000 inhabitants) occurred in the year 1998 [46]. In 1999, 50% of Brazilian municipalities had already reported DF cases and* Ae. aegypti* had been detected in 64% of them and only the states of Acre and Distrito Federal did not document cases of dengue [52].

DENV-3 first appeared and caused an outbreak in the state of Rio de Janeiro in December 2000 [58] (Figure 3), with 3,220 dengue cases reported. In 2002, DENV-3 caused one of the largest outbreaks ever reported in Brazil (696.472 cases/100,000 inhabitants) in the state of Rio de Janeiro, with 288,245 reported cases and 91 deaths [47, 52, 53, 59, 60].

Between 1981 and 2006, 4,243,049 dengue cases were reported in Brazil, including 5,817 cases of SD and 338 fatalities. The highest number of reports came from the Northeast and Southeast regions [59]. One of the states of the Northeast region, Pernambuco, had from 1995 to 2006 approximately 380,000 DF cases, 612 DHF cases, and 33 deaths reported [61].

From 2002 to 2006, the most prevalent serotype in Brazil was DENV-3. In the following years, between 2007 and 2009, DENV-2 was responsible for the majority of cases [62]. From 2000 to 2007, Brazil alone accounted for more than 60% of the world reported cases of dengue. Dengue epidemics occurred in different regions of the country and cases of DENV-1, DENV-2, and DENV-3 were reported in all states [57, 63, 64].

Between 2007 and 2008, DENV-2 caused an intense outbreak in the state of Rio de Janeiro with a higher number of severe cases (954 and 15,730, resp.) and fatalities (41 and 263, resp.) than previous outbreaks, primarily among children and adolescents [6, 64]. In these 2 years, 851 deaths due to severe dengue were registered in the country [46]. In 2008, approximately 80% of the cases reported in the country occurred in the Southeast and the Northeast regions [62]. In 2009-2010, over a million suspected cases of DF and 665 deaths were reported in Brazil, with DENV-1 accounting for most of the cases [62]. However, in 2009 in the state of Espírito Santo in the Southeast region, DENV-2 was the predominant type between the 53,708 cases notified, followed by DENV-1 [65].

The 2010 dengue epidemic in Brazil was characterized by several outbreaks in 21 states, Rio Grande do Sul, Santa Catarina, Paraná, São Paulo, Minas Gerais, Rio de Janeiro, Espírito Santo, Bahia, Mato Grosso, Mato Grosso do Sul, Tocantins, Acre, Pará, Roraima, Goiás, Rondônia, Alagoas, Pernambuco, Rio Grande do Norte, Piauí, and Ceará, and by cocirculation of all serotypes, with DENV-4 reemerging in the northern region of the country after 28 years of absence [66] (Figure 3, Table 2). Thereafter, DENV-4 was reported in the states of Amazonas, Amapá and Pará [67], São Paulo [68], and Rio de Janeiro [69]. Currently, 100% of dengue cases in the states of Rondônia, Amazonas, Piauí, and Paraíba, all with incidence rate over 100 cases/100,000 inhabitants, are due to DENV-4. Overall, in Brazil the current most prevalent DENV types are DENV-1 (83.3%), followed by DENV-4 (15.1%), DENV-2 (1.3%), and DENV-3 (0.3%) [46].

### 2.2. Current Situation

Epidemiologic studies performed in Brazil during 2005 and 2008 demonstrated that dengue affected predominantly adults and usually occurred in cities with more than 500,000 inhabitants [47, 60]. Nevertheless, the profile of dengue outbreaks in Brazil is changing and current data underscore a different epidemiological pattern, wherein an increased proportion of severe cases occur among children [70, 71] and in low population density areas [52]. During the 2007 epidemic, 40% of dengue cases came from municipalities with fewer than 100,000 inhabitants [52], and more than 50% of cases were in children younger than 15 years of age [70]. The same pattern occurred in Ceará state: in 2007 the majority of hospitalized cases due to SD were in children <15 years of age; in 2008, DF incidence was highest (599.4 cases/1000,000 inhabitants) among children <10 years of age [71].

**Figure 4:**
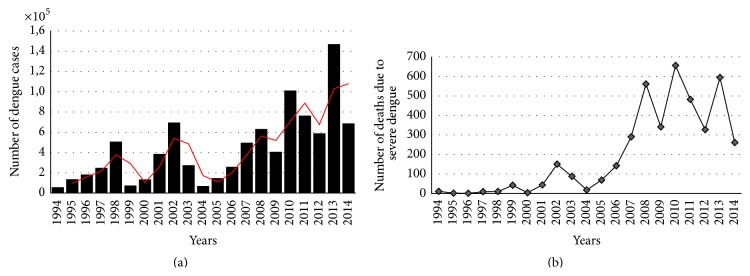
Number of cases (a) and deaths (b) due to confirmed dengue cases in Brazil from 1994 to 2014. The numbers were obtained from Brazilian Ministry of Health website, accessed at http://portalsaude.saude.gov.br/. Dengue cases were confirmed by laboratory tests as described in Introduction. Red line represents two periods moving average and was set up with the Spreadsheet Software Program Microsoft Excel.

Between the years 2000 and 2006, the ratio of DF/SD cases reported in Brazil was 467.7, considerably higher than that reported during the same period in Honduras, Venezuela, Colombia, and Mexico, becoming the highest ratio in the continent [72]. However, the lethality ratio of dengue in Brazil was 0.01% and mortality per 100,000 inhabitants was 0.03, both from 1995 to 2009 [45]. Still, the diagnoses of severe dengue, the number of hospitalized cases, and the number of deaths attributed to dengue have increased during the past 10 years [47] (Figure 4).

When compared to 2012, there was a decrease in the number of cases compared with 2011 but in 2013 the incidence increased by 190% [73]. According to this data, in 2013, 1,468,873 cases were reported in Brazil, of which 6,969 were severe cases, with 545 deaths [46]. In 2014, the Brazilian Ministry of Health had reported to PAHO 591,080 cases of dengue for an incidence rate of 291.5/100,000 inhabitants and 410 deaths. It represents a decrease of 39% of deaths in relation to the same period in 2013 [44, 46]. As of April 2015, the Brazilian Ministry of Health has registered 1,254,907 notified cases of dengue, representing an increase which is more than twofold compared to that of 2014, and it is being considered a new epidemic in the country, with 530 deaths due to SD (53% more compared to the same period of 2014) [46].

The observed clinical manifestations of dengue may include neurological, hepatic, and cardiac involvement as described in Brazil and other countries of the Americas and in Southeast Asia [52]. Neurological manifestations of DENV include reduced levels of consciousness, severe headaches, neck stiffness, focal neurological signs, tense fontanels, and convulsions [74]. The pathophysiology is attributed to factors, such as cerebral edema, cerebral hemorrhage, hyponatremia, and fulminant hepatic failure with encephalopathy, cerebral anoxia, microcapillary hemorrhage, and release of toxic products [23]. Some authors state that dengue infection should be considered a possible cause of encephalitis in endemic regions [74, 75]. During the outbreaks of 1997 and 2002, there were reports of 41 cases with neurological manifestations in the state of Pernambuco. Encephalitis, Guillain-Barré syndrome, convulsions, meningoencephalitis, and reduced levels of consciousness were the most common manifestations [76].

Severe dengue is not necessarily associated with secondary infections. Disease severity appears to be determined by many risk factors, including the strain virulence and host immunity [10, 52, 60]. Concerning the differences of severity between Brazilian individuals, the human leukocyte antigen (HLA) gene, which mainly encodes the major histocompatibility complex (MHC) class I and class II molecules, has been appointed as a possible marker of susceptibility to dengue disease. A recent study of dengue patients in Recife, Brazil, showed a significant association of MHC I and MHC II molecules encoding the alleles HLA-B^*∗*^44, -B^*∗*^50, and -DR^*∗*^16 with increased susceptibility to SD [77]. The link between HLA class I alleles and SD was found among patients from Rio de Janeiro with association of SD with the HLA-A^*∗*^01 allele and a potential protective role of the HLA-A^*∗*^ allele in SD [78].

### 2.3. The Different Genotypes of* Dengue Virus* in Brazil

In addition to frequent outbreaks, an increased genetic diversity of DENV may have contributed to severe consequences such as an increase in pathogenicity, transmissibility, and virulence properties [79]. Genetic diversity allows enhancement of viral replication following heterologous infections due to limited cross-reactive immunity [80]. The four DENV types are known to be genetically distinct from each other, and each one has genetic variations classified as subtypes or genotypes [81].

DENV-1 has five distinct genotypes, designated I to V [62, 80]. The genotype V of DENV-1 was identified in the years 2009-2010 in the states of Rio de Janeiro and Espírito Santo, in Southeast of Brazil. The circulating strains of genotype V were grouped into a clade (lineage II) that was distinct from the clade represented by earlier Brazilian DENV-1 strains (lineage I). Another distinct clade (lineage III), identified in samples from Rio de Janeiro in 2010 and 2011, showed similarity to strains isolated in 2007 and 2008 in Colombia, Venezuela, and Mexico [62].

DENV-2 comprises six genotypes: Asian I, Asian II, Southeast Asian/American, Cosmopolitan, American, and Sylvatic genotypes [62, 65, 81]. The Sylvatic genotype represents strains from humans, arboreal mosquitoes, and nonhuman primates collected in West Africa and Southeast Asia [80]. Strains from the Southeast Asian/American genotype were isolated during epidemics periods in 1990 and 1998 in Rio de Janeiro [26, 82]. In 2010, DENV-2 isolates circulating in the cities Guarujá and Santos, in the state of São Paulo, clustered within the Southeast Asian/American genotype. This is the same genotype that circulated in Rio de Janeiro in 2007-2008 and in Espírito Santo in 2009 [26, 65, 82, 83]. These observations suggest that the genetically different viruses detected in Rio de Janeiro could have resulted from local evolution of DENV-2 since its introduction in 1990 [82, 83]. Indeed, DENV-2 strains circulating in Brazil belong to separate Southeast Asian/American genotype lineages: lineage I, circulating from 1990 to 2003, and lineage II for strains isolated after 2007 [83].

DENV-3 has five genotypes designated I to V by phylogenetic analysis based on different viral gene regions [80, 84]. The majority of Brazilian samples are grouped with genotype III [85] but genotype I was isolated between 2002 and 2004 in the state of Minas Gerais and later detected in* Ae. aegypti* mosquitoes and eggs [86]. This genotype was associated with a fatal case [87]. DENV-3 strains isolated during 2006 and 2007 outbreaks in the city of São José do Rio Preto in the state of São Paulo fell within genotype III and grouped with Brazilian isolates from different regions in different years, including samples from Acre in 2004 and Rio de Janeiro in 2002 [85]. Four distinct lineages of DENV-3 genotype III have been identified in Brazil (I to IV); however only lineages I and II seem to have become effectively established and disseminated in the country [88].

DENV-4 has four genotypes: I to IV, where genotype IV is the only Sylvatic DENV-4 strain isolated from sentinel monkeys in Malaysia [80, 89]. Genotype II predominates in Brazil. It was introduced into the Americas (Caribbean region) around 1978 [67]. Sequence analyses performed with samples of DENV-4 from São Paulo and Rio Grande do Sul revealed that all samples were of genotype II and grouped with samples from the Caribbean and northern South America [90]. In addition, genomic and envelope protein phylogeographic analyses showed that DENV-4 genotype I was isolated in 2011 in the city of Salvador, in the state of Bahia, and appeared to originate from mainland Southeast Asia [67]. DENV-4 genotype I infecting* Aedes aegypti* have been also described in the city of Manaus, in the state of Amazonas [91].

Introduction of new DENV genotypes may have facilitated the increase in clinical severity of dengue infections observed in more recent dengue epidemics [81].  Table 3 shows the DENV genotypes known to circulate in Brazil.

### 2.4. Dengue Vectors in Brazil

Dengue is transmitted mainly by* Ae. aegypti* and* Ae. albopictus*. Both vectors are adapted to the peridomestic environment where they feed on humans and domestic animals and oviposit in a variety of natural and artificial water holding containers [86, 92–94].

The efficiency of transmission depends on vector competence which is defined as the susceptibility of a mosquito to become infected and subsequently transmit the virus through the bite [95].* Ae. albopictus* is more susceptible than* Ae. aegypti* to midgut infection by DENV. However, a smaller proportion of* Ae. albopictus* develops disseminated infection when compared to* Ae. aegypti*, suggesting that DENV dissemination by* Ae. albopictus* is less efficient [93].

Between the 1950s, 1960s, and most of the 1970s, epidemic dengue was rare in Central and South America because* Ae. aegypti* had been eliminated from most of the countries in the continent. The eradication program organized by PAHO was discontinued in the early 1970s, and the mosquito was reintroduced in countries from which it had been previously eradicated [96].

In Brazil,* Ae. aegypti* has been responsible for dengue transmission since the early 1980s.* Ae. albopictus* was introduced in Brazil in 1986 [93, 94] and is present in all Brazilian states [97].* Ae. albopictus* is not considered as a vector of DENV in the country and has not been associated with dengue epidemics [93, 97, 98]. However, the occurrence of vertical transmission of DENV-2 and DENV-3 in* Ae. aegypti* and* Ae. albopictus* has already been observed in Fortaleza [94] and raises questions about the potential for transmission of DENV by* Ae. albopictus* in Brazil. Indeed, studies in the state of Rio de Janeiro have shown that* Ae. albopictus* was the dominant species in discarded tires used as traps [99, 100].

The continuous global expansion of* Ae. albopictus* is a serious concern as it may play a role in the maintenance of DENV in nature [95] and alter the transmission dynamics of many arboviral diseases increasing the risk of mosquito-borne viral infections among humans [101].

A major example is Chikungunya fever (CHIK), a disease caused by an arthropod-borne virus, the* Chikungunya virus* (CHIKV), which often cocirculates with DENV in their respective endemic regions. CHIKV reached the Americas in December 2013, causing outbreaks that now affect 44 territories throughout the Americas with more than 1.2 million suspected cases reported to PAHO [102, 103]. More than 1,000,000 suspected cases, 24,375 laboratory-confirmed cases, and 178 deaths were reported to PAHO between 2013 and 2014 [102]. The Brazilian government has reported three imported cases of CHIK in 2010 [104]. 3,195 CHIK autochthonous cases have been reported in Brazil from which 2,196 were confirmed as of January 2015 [46].

Dengue epidemics causing mostly dengue fever attributed to transmission by* Ae. albopictus* have occurred in Asia (Japan, China, Maldives Islands, and northern Taiwan), Africa (Seychelles Islands), Oceania (La Reunion Island), and Hawaii. Major epidemics of severe dengue have only occurred in areas where* Ae. aegypti* is found [93].

Many factors support proliferation of* Ae. aegypti* and consequently the sustained transmission of mosquito-borne diseases in Brazil, including the climate, high human population density in large cities, precarious socioeconomic status, and lack of infrastructure, particularly adequate sanitation [52, 72]. Meteorological conditions and seasonal variations may affect the distribution and abundance of the vector [72]. As an example, rainfall affects mosquito abundance positively through the creation of new breeding sites [105]. The vector in turn is able to adapt to new environmental situations that affect dengue epidemiology [52].

An initial report of* Ae. aegypti* occurrence in Brazil in 2014 shows the following percentage of municipalities at risk, based on the Breteau Index (density of mosquito larvae): 32.7% in the North, 34.3% in the Northeast, 8.4% in the Southeast, 5.8% in the Midwest, and 32.5% in the South region [46]. It is important to observe that, even with a low percentage of municipalities at risk, the Southeast region presented a dengue incidence rate of 366.9 cases/100,000 inhabitants in 2014 (Figure 2). The appropriateness of larval indices for population monitoring has been questioned because their relationship with adult* Aedes* densities heavily depends on larval mortality [106, 107], but the Breteau Index continues to be used by the Brazilian Ministry of Health.

### 2.5. Prevention and Research

No safe and effective vaccine for dengue is currently available. Therefore, the control of DENV infections relies solely on vector control. In this context, some prevention measures have been adopted in Brazil, such as development and implementation of public awareness campaigns to educate the population with the aim of reducing the availability of* Ae. aegypti* breeding sites. Insecticide application [52] and monitoring systems of* Aedes* larvae and eggs, as well as mosquito adults, have also been used [108, 109].

Health professionals in the country are being trained to improve early diagnosis and treatment of severe dengue [52]. Indeed, an effective diagnostic test contributes significantly to the clinical treatment, etiologic investigation, and control of DENV infections [42]. Since 2002, Brazil has adopted a clinical protocol looking at the clinical progression of disease. It is based on the recognition of clinical and laboratory data and conditions related to severity, with the goal of appropriate treatment and avoidance of deaths. In this context, the Brazilian Ministry of Health recommends virus isolation and serology as the main diagnostic tests [110].

Currently, a new vector control strategy has been developed, which consists of the introduction of* Wolbachia* bacteria into target vector populations, which directly inhibits the ability of several pathogens to infect* Ae. aegypti* [111–113]. Another advanced technique developed by Oxitec is the releasing of transgenic mosquitoes to reduce wild mosquitos numbers [114, 115]. In Brazil, the evaluation of Oxitec mosquitoes is called “Project Aedes Transgenico” (PAT) [116]. Around 11 million male mosquitoes have been released in the country from February 2011 through February 2012 [116, 117]. Results of both strategies have been encouraging. However, more studies are needed before considering these approaches safe and effective.

National and international efforts have been applied to the development of a dengue vaccine. The major challenge is to create a safe and effective tetravalent vaccine that generates immunity to all four serotypes. Patients that recover from dengue infection by one serotype are at risk of developing severe dengue when infected subsequently by a different serotype. The concern about a vaccine unable to raise immunity to the four serotypes simultaneously is that it may increase the risk of severe dengue when the immunized individual is infected by a serotype for which the individual is not immune [52, 118]. One obstacle that hampers research in this area is the lack of an adequate animal model for SD [15].

Several vaccine candidates are currently being evaluated in clinical trials and vaccines are likely to be available within the next several years [119–121]. A recent cost-effectiveness study of dengue vaccines in Brazil showed that herd-immunity may be achieved by vaccinating 82% of the population at a vaccine efficacy of 70%. At this efficacy, vaccination would cost up to US$534 per vaccinated individual with cost-savings of up to $204 [120]. These values indicate that, even at a relatively low efficacy, vaccination would still be cost-effective since the total vaccination cost would be sufficiently low [120]. Indeed, the vaccination in low- and middle-income countries brings important economic benefits and cost-effectiveness studies suggest that vaccines are an efficient public health investment [122]. Another detailed economic analysis of the steady-state production of 60 million doses per year at the Instituto Butantan in the state of São Paulo showed that the vaccine, if it proves to be safe and effective, can be available at a price that most ministries of health in developing countries could afford [123].

## 3. Conclusions

Dengue is considered the most important mosquito-borne viral disease in the world by the World Health Organization [42] and an ongoing threat to the Brazilian population. Because of the lack of vaccines and specific therapies, vector control is currently the only effective measure available to control the spread of the disease. Many studies are being conducted to develop and to improve technologies that are able to reduce or eliminate* Ae. aegypti* populations on a large scale. However, the constant occurrence of dengue epidemics in Brazil demonstrates that sustained dengue control and surveillance policies at the local level (municipalities) are still needed. It will avoid the constant reestablishment of foci of active mosquito breeding and transmission of the infection giving rise to new cases of the disease.

The tropical climate makes Brazil susceptive to cocirculation of different arboviruses, such as DENV, CHIKV, and the recently introduced Zika virus, already reported in several Brazilian states. Those viral infections are oligosymptomatic and clinically similar, hampering the differential diagnosis. Furthermore, there is still a need of improvement of laboratory diagnosis for dengue, since serologic tests available often take several days to be completed and present high cross-reactivity among DENV serotypes. Beyond the differentiation between the arboviruses cocirculating, efficient diagnosis allows appropriate patient care, generation of accurate epidemiological data, and implementation of effective public health interventions.

## Figures and Tables

**Table 1:** Dengue cases and DENV serotypes reported in Brazil in 2014.

Geographical regions and states	Number of inhabitants^a^	Number of cases reported^b^	Serotypes confirmed^b^
Southeast region	85,505,375	312,318	
Minas Gerais (MG)	20,813,172	59,222	DENV-1, DENV-3, and DENV-4
Espírito Santo (ES)	3,906,648	19,223	DENV-1, DENV-4
Rio de Janeiro (RJ)	16,520,687	7,823	DENV-1, DENV-4
São Paulo (SP)	44,264,868	226,040	DENV-1, DENV-2, and DENV-4
Midwest region	15,344,314	114,814	
Mato Grosso do Sul (MS)	2,638,452	3,594	DENV-1, DENV-2, and DENV-4
Mato Grosso (MT)	3,247,377	7,232	ND
Goiás (GO)	6,574,219	92,311	DENV-1, DENV-4
Distrito Federal (DF)	2,884,266	11,677	DENV-1
Northeast region	41,228,546	90,192	
Maranhão (MA)	6,876,025	2,416	ND
Piauí (PI)	3,200,725	7,665	DENV-1
Ceará (CE)	8,883,739	22,974	DENV-1, DENV-3, and DENV-4
Rio Grande do Norte (RN)	3,428,430	11,285	DENV-1, DENV-2, and DENV-4
Paraíba (PB)	3,957,858	5,575	DENV-1, DENV-2, DENV-3, and DENV-4
Pernambuco (PE)	9,315,660	10,446	DENV-1, DENV-2, DENV-3, and DENV-4
Alagoas (AL)	3,332,654	13,275	ND
Sergipe (SE)	2,233,455	2,275	DENV-1, DENV-4
Bahia (BA)	15,170,625	14,281	DENV-1, DENV-4
North region	17,367,554	49,534	
Rondônia (RO)	1,760,186	2,104	DENV-1, DENV-4
Acre (AC)	797,676	28,931	DENV-1
Amazonas (AM)	3,910,647	6,472	DENV-4
Roraima (RR)	503,116	1,181	DENV-1, DENV-2, DENV-3, and DENV-4
Pará (PA)	8,127,744	4,833	DENV-1, DENV-2, and DENV-4
Amapá (AP)	760,634	1,958	DENV-1
Tocantins (TO)	1,507,551	4,055	DENV-1, DENV-4
South region	29,131,203	24,222	
Paraná (PR)	11,133,587	23,924	DENV-1, DENV-4
Santa Catarina (SC)	6,758,785	141	ND
Rio Grande do Sul (RS)	11,238,831	157	DENV-1, DENV-4

Number of dengue cases and DENV serotypes reported in Brazil in 2014 by the Ministry of Health. Data are organized by geographical regions and the states they include. ^a^Data obtained in http://www.ibge.gov.br/apps/populacao/projecao/index.html (last accessed on January 30, 2015). ^b^Data reported by Brazilian Ministry of Health until the epidemiological week 53—12/28/14 to 01/03/15. Source: http://portalsaude.saude.gov.br (last accessed on May 20, 2015). ND: not determined.

**Table 2:** Dengue activity in Brazil between 1845 and 2010.

Year (s)	Activity reported	Dengue serotype	Location	Reference
1845	1st dengue epidemic was reported	Unknown	Rio de Janeiro	[46]

1981	1st dengue epidemic in Brazil after *Ae*. *aegypti* reinfestation	DENV-1 DENV-4	Roraima	[46, 50]

1986-1987	Epidemic	DENV-1	Rio de Janeiro	[6, 48, 49, 51]

1990	First identification of DENV-2	DENV-2	Rio de Janeiro	[26, 48, 49, 54]

1990–2000	DENV spread intensified contributing to several outbreaks	DENV-1 DENV-2	Southeast and northeast region	[47, 55, 56]

2000	1st appearance of DENV-3 in Brazil	DENV-3	Rio de Janeiro	[48–51, 53]

2002	One of the largest dengue outbreaks in Brazil since the virus emergence	DENV-3	Rio de Janeiro	[48–51]

2000–2007	Brazil reported >60% of the cases registered in the world	DENV-1 DENV-2 DENV-3	All Brazilian states	[47, 54, 55]

2007-2008	Intense outbreak with high number of severe cases and fatalities	DENV-2	Rio de Janeiro	[6, 55]

2009	Large outbreak	DENV-2	Espírito Santo	[56]

2010	Several outbreaks	DENV-1 DENV-2 DENV-3 DENV-4	21 Brazilian states	[26]

2010	Reemergence of DENV-4	DENV-4	Roraima, Amazonas, Amapá, Pará, São Paulo, and Rio de Janeiro	[57, 58, 65, 68]

The paper text contains more detailed information about the outbreaks and other epidemics description as well.

**Table 3:** DENV genotypes identified in Brazil.

DENV serotype	Genotype circulating in Brazil	Reference
DENV-1	Genotype V	[53, 68]
DENV-2	Southeast Asian/American	[26, 56, 71, 72]
DENV-3	Genotypes I and III	[60, 74–76]
DENV-4	Genotypes I and II	[57, 78, 90]
